# Clinical and immunological relevance of anti-neuronal antibodies in celiac disease with neurological manifestations 

**Published:** 2015

**Authors:** Giacomo Caio, Roberto De Giorgio, Alessandro Venturi, Fiorella Giancola, Rocco Latorre, Elisa Boschetti, Mauro Serra, Eugenio Ruggeri, Umberto Volta

**Affiliations:** *Department of Medical and Surgical Sciences, University of Bologna and St. Orsola-Malpighi Hospital, Bologna, Italy *

**Keywords:** Celiac disease, Neurological symptoms, Anti neuronal antibodies

## Abstract

**Aim:**

To assess anti-neuronal antibodies (NA) prevalence and their correlation with neurological disorders and bowel habits in celiac disease (CD) patients.

**Background:**

Neurological manifestations are estimated to occur in about 10% of celiac disease patients and NA to central nervous system (CNS) and enteric nervous system (ENS) are found in a significant proportion of them. Little is known about the clinical and immunological features in CD patients with neurological manifestations.

**Patients and methods:**

NA to CNS and ENS were investigated in 106 CD patients and in 60 controls with autoimmune disorders by indirect immunofluorescence on rat / primate cerebellar cortex and intestinal (small and large bowel) sections.

**Results:**

IgG NA to CNS (titer 1:50 - 1:400) were positive in 23 celiacs (21%), being more frequently detected in those with neurological disorders that in those without neurological dysfunction (49% vs. 8%, P< 0.0001). Of the 26 celiacs (24%) with IgG NA to ENS, 11 out of 12 with an antibody titer > 1:200 had severe constipation. Only one patient with cerebellar ataxia and intestinal sub-occlusion was positive for NA to CNS and ENS. NA to CNS and ENS were found in 7% and 5% of controls, respectively.

**Conclusion:**

In CD the positivity of NA to CNS can be regarded as a marker of neurological manifestations. High titer NA to ENS are associated with severe constipation. The demonstration of NA to CNS and ENS suggests an immune-mediated pathogenesis leading to central neural impairment as well as gut dysfunction (hence constipation), respectively.

## Introduction

 Celiac disease (CD) is a chronic, multisystemic, autoimmune disorder, triggered by gluten ingestion, in genetically sensitive patients (1). Further to the small bowel, the main target organ, CD is recognized to be a systemic disorder involving many other tissues and organs, e.g. skin, thyroid, liver, joints, muscle, bone, pancreas and nervous systems (2). The reasons for the multisystemic nature of CD is ascribable to the widespread localization of the main autoantigen of the disease, i.e. tissue transglutaminase type 2 (TG2) and related isoforms (TG3 and TG6) (2). Idiopathic cerebellar ataxia (ICA), peripheral neuropathy (PN) and different forms of epilepsy represent the most common neurological manifestations occurring in CD patients (3-5). Other CD-associated neurological diseases include multiple sclerosis, migraine, multifocal leukoencephalopathy, dementia, chorea, and attention / memory impairment. Taken together, neurological manifestations can occur in about 10% of CD patients.

The pathogenesis of neurological involvement in CD is still debated. Previous pathogenetic theories regarded mainly vitamin deficiencies (e.g. thiamine, folic acid, cyanocobalamin, vitamin E), due to intestinal malabsorption (6). More recently, however, pathologic data on the CNS of patients with neurological CD, indicated that immune-mediated mechanisms can play a role by evoking neuronal injury and dysfunction (7). In this line, circulating anti-neuronal antibodies (NA) of the IgG class have been demonstrated to target central and enteric nervous systems (CNS and ENS, respectively) in a significant proportion of neurological CD patients (4).

This study has been designed to gain further insights about clinical and immunological features of CD patients with neurological manifestations. In particular, we aimed to assess the prevalence of NA to CNS and / or ENS. Another objective of this study is to expand the knowledge of NA to ENS and explore their association with gut dysfunction. 

## Patients and Methods

A total number of 106 non consecutive patients with untreated CD (24 males, 82 females; age range 17-59 years) were enrolled in this study. Of these, 35 CD patients (5 males, 30 females; age range 19-56 years) had neurological manifestations (11 cases of ICA; 10 epilepsy without cerebral calcifications at computed tomography; 4 multiple sclerosis, MS; 5 attention / memory impairment syndrome, AMIS; and, finally, 5 PN). CD patients (n= 71; 19 males, 52 females; age range 17-59 years) without neurological manifestations were also assessed. In all cases, in addition to positive serology (anti-tTG2 and anti-endomysial antibodies), the diagnosis of CD was always confirmed by endoscopic duodenal biopsy revealing villous atrophy (Marsh III lesion) (8, 9).

As a control group, 60 patients with immune-mediated disorders (6 Crohn disease; 4 ulcerative colitis; 15 autoimmune hepatitis; 15 primary biliary cirrhosis; and 10 CREST syndrome) were investigated. None of them showed any signs of neurological manifestations. All patients and controls gave their informed consent before entering the study. Since patients were not individually identified, a simplified International Review Board approval by the Ethics Committee of the St. Orsola Malpighi Hospital was obtained.

***Detection of NA***

NA to CNS and ENS were detected by indirect immunofluorescence in all CD and control patients. Cryostat sections (5 mm) of monkey and rat cerebellum (for NA targeting the CNS) as well as rat ileum and colon (for NA targeting the ENS) (Astra srl, Milano, Italy) were used. Sera of patients were tested at the initial dilution of 1:10 and, when positive, titrated to the end point. Rabbit anti human IgG (Dako, Copenhagen, Denmark) were used as secondary antibody at the appropriate working dilution (1:60 and 1:100 on rat and monkey tissue, respectively).

***Statistical analysis***

The two-tailed Fisher's exact test was used to compare the clinical findings and prevalence of NA to CNS / ENS in CD patients with and without neurological manifestations vs. autoimmune disorder controls. 

## Results

**Figure 1:**
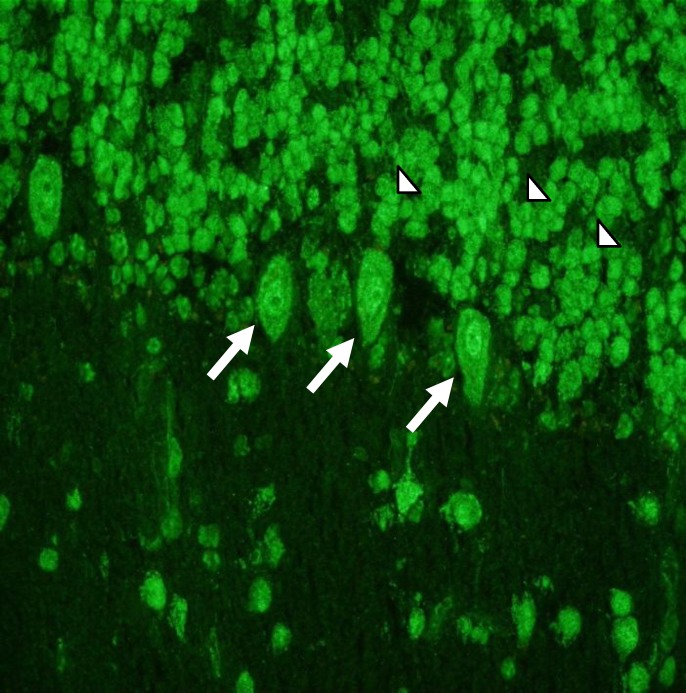
Immunofluorescent pattern of NA to CNS on rat cerebellum sections. Note the intense immunostaining in the nucleus and cytoplasm of Purkinje cells (arrows) along with positivity of granular layer neurons (arrowheads) in a CD patient with cerebellar ataxia. Original magnification 40x.

The immunofluorescent pattern of NA to CNS was characterized by an intense immunostaining in the nucleus and cytoplasm of Purkinje cells along with positivity of granular layer neurons on rat and monkey cerebellum sections (Figure 1). 

**Figure 2:**
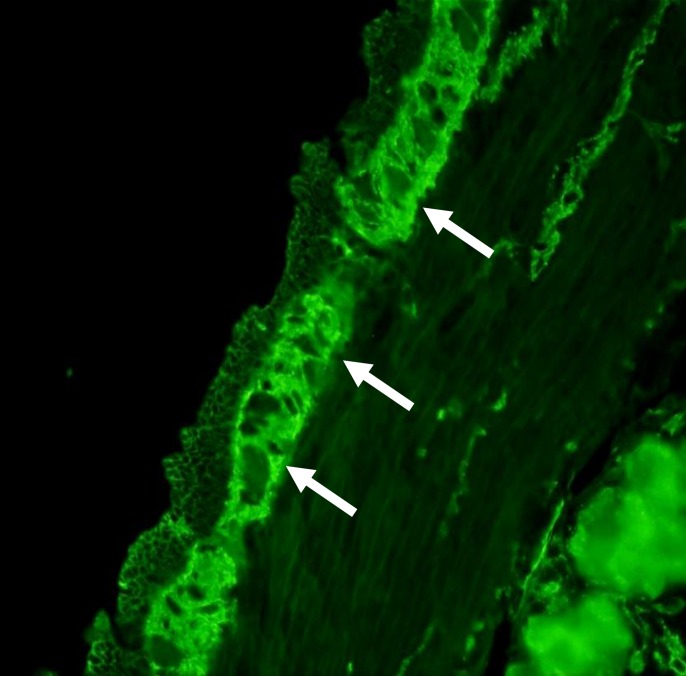
Immunofluorescent pattern of NA to ENS on rat ileum sections. Note the bright staining in the myenteric plexus (arrows) of the rat ileum observed in a CD patient with severe constipation. Magnification 40x

**Figure 3:**
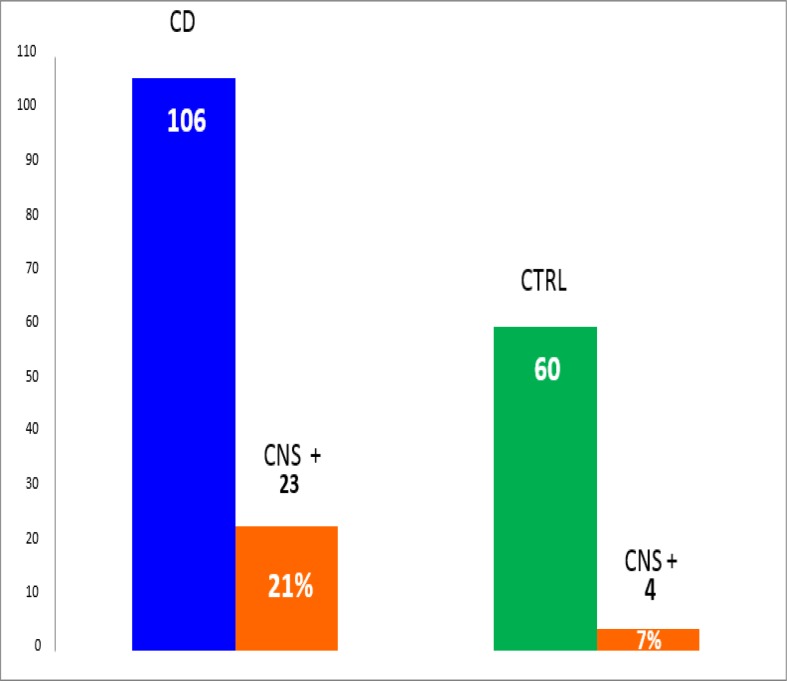
Prevalence of NA to CNS in the whole group of celiac disease patients vs. autoimmune controls (P*<*0.05, two-tailed Fisher’s exact test). Abbreviations: CD: Celiac disease; CNS+: NA to CNS; CTRL: controls.

A bright staining in the myenteric and submucosal plexuses of the rat ileum and colon represents the typical pattern of NA to ENS (Figure 2). NA to CNS were found in 23 (21%) of the whole group of the 106 CD patients in comparison with their positivity in 4 (7%) of the 60 controls with autoimmune disorders (P< 0.05 (Figure 3). 

**Figure 4:**
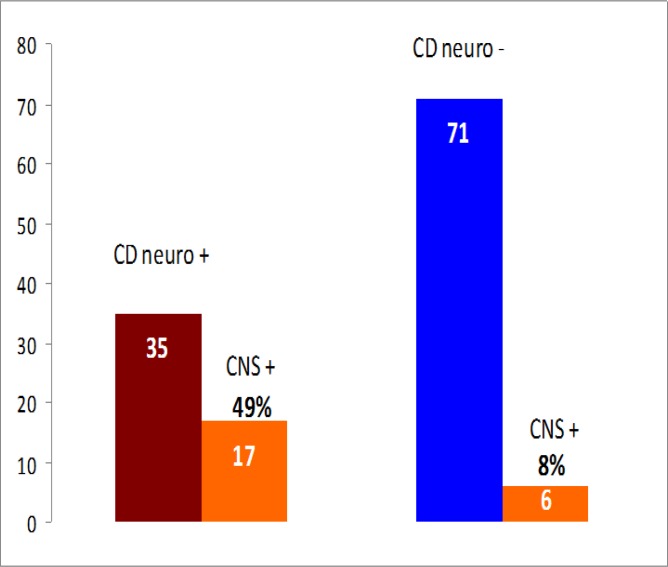
Prevalence of NA to CNS in neurological CD vs. non-neurological-CD (P<0.0001, two-tailed Fisher’s exact test). Abbreviations: CD neuro+: neurological celiac disease; CD neuro-: non-neurological celiac disease; CNS+: NA to CNS.

NA to CNS were detected in 17 (49%) of the 35 patients with neurological CD vs. 6 (8%) of the 71 patients with non-neurological CD patients (*P*< 0.0001) (Figure 4). Of the 17 neurological CD patients with NA to CNS, 7 had ICA, 4 epilepsy, 2 MS, 2 PN and 2 AMIS. None of the 4 controls with immune-mediated disorders positive for NA to CNS showed neurological manifestations.

**Figure 5:**
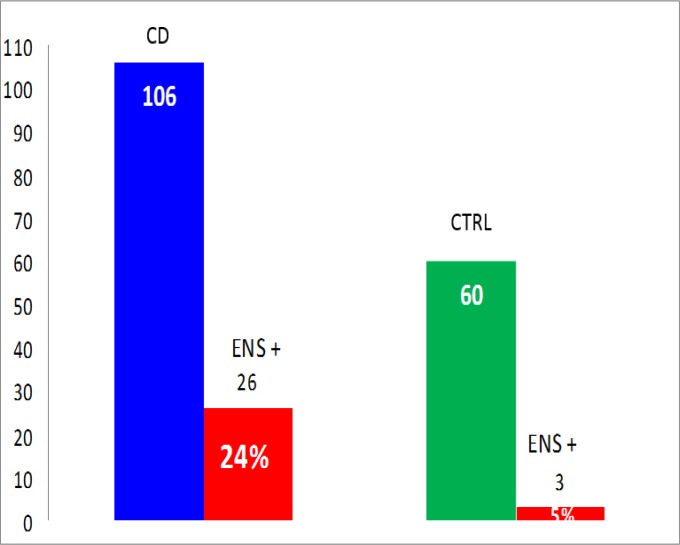
Prevalence of NA to ENS in the whole group of celiac disease patients vs. autoimmune controls (P*<*0.05, two-tailed Fisher’s exact test). Abbreviations: CD: celiac disease; ENS+: NA to ENS; CTRL: controls.

**Figure 6:**
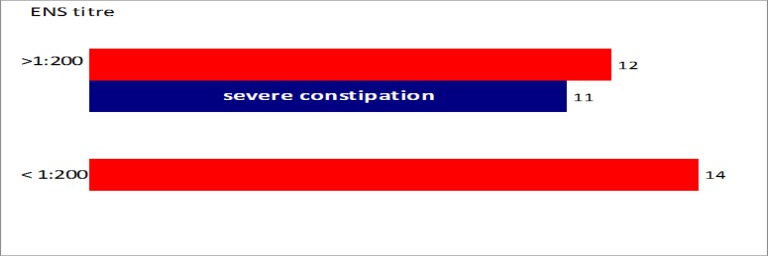
Correlation between antibody titer of NA to ENS and severe constipation: 11 of 12 CD patients with antibody titer > 1:200 showed severe constipation (P*<* 0.0001, two-tailed Fisher’s exact test). Abbreviations: "ENS titre" refers to titer of NA to ENS.

NA to ENS were positive in 26 (24%) of the 106 CD patients vs. 3 (5%) of the 60 controls with autoimmune disorders (P< 0.05) (Figure 5). NA to ENS were not significantly different in neurological vs. non-neurological CD, being positive in 9 (26%) of the 35 vs. 17 (24%) of the 71 patients, respectively. A high antibody titre (> 1:200) of NA to ENS was detected in 12 (46%) of the 26 CD patients and, notably, 11 of these 12 complained of severe, Rome III-defined constipation (P< 0.0001) (Figure 6). Only one patient with cerebellar ataxia and recurrent intestinal sub-occlusive episodes was positive for NA to CNS and ENS. 

## Discussion

The link between CD and neurological disorders has been established since many years and neurological impairment can be often the only clinical manifestation for suspecting CD (2-7). A CD antibody screening should always be sought in any patients with ICA, PN and, especially, in those cases with pharmacologically resistant forms of epilepsy. The identification of gluten-sensitive enteropathy and the subsequent strict gluten free diet can often result into the improvement of neurological impairment (3, 7). Published data showed that ICA and PN are the most common neurological disorders being found in 2-15% and 1.5-8% of CD patients, respectively (10, 11).

The present study was designed to test the prevalence of NA to CNS and ENS in patients with CD-related neurological manifestations. Our results demonstrated that almost half of neurological CD patients had circulating NA to CNS mainly detected in patients with ICA. Also, NA to CNS were found in patients with CD-related epilepsy, PN, MS, and, finally, AMIS, findings that confirm previously published data (4). In contrast, likewise autoimmune disorders, patients with non-neurological CD showed a very low prevalence of these autoantibodies, thus strengthening a significant association between NA to CNS and neurological CD (4).

Further to NA to CNS, we found positive NA to ENS in about a fourth of the total (n= 106) CD group with a significantly higher prevalence than that of autoimmune controls. Although NA to ENS showed a similar prevalence in neurological and non-neurological CD, their detection was highly associated with gut dysfunction. In particular, a subset (11 / 12) of CD patients showing high titer (> 1:200) of NA to ENS had a very severe form of chronic constipation as established by Rome III criteria. 

 Constipation is the prototype of functional bowel disorders and its occurrence has been estimated in 15-20% of the general population. 

Based on this high prevalence one cannot discard the possibility that constipation may be just a coincidence in our CD patients. However, the present data highlighted a strong association linking CD to chronic constipation. Indeed, the ENS is one of the major systems controlling gut physiology. Hence any noxa (i.e. NA) perturbing ENS morpho-functional integrity may cause bowel dysfunction (dysmotility, altered secretion) known to underlie constipation. Compared to CD patients without NA to ENS, sera of CD patients containing NA to ENS exposed to neuronal cultures evoked apoptosis and neuronal loss (7). Also, previous data demonstrated that NA may alter the ascending reflex of small bowel peristalsis and inhibit motorneuron excitability in vitro (12). Taken together these results provide a pathophysiological basis to the concept that autoimmunity targeting ENS can be an important mechanism operating in chronic constipation identifiable in CD patients. Similarly to ENS, also NA to CNS can exert a pathogenetic potential on a wide array of different central and peripheral neurons, thereby leading to neurological manifestations. In support of this role, previous pathological data showed an immune (humoral and cellular) infiltrate in the CNS (mainly cerebellum) of patients with neurological impairment associated to CD (13).

The reasons to explain the immune mediated targeting of enteric, peripheral and central neurons are still partially understood. One possibility is that tissue TG isoform expression may drive an activation of the immune system in susceptible CD patients. Indeed, one of these autoantigen can be the TG6 isoform and anti-TG6 antibodies have been identified in patients with ICA and PN with or without underlying CD (14). Whether the NA to CNS or to ENS may target or cross react with TG6 (or other TG isoforms) remain to be elucidated. Moreover, the presence of high titer anti-gliadin antibodies (AGA) has been demonstrated in patients with neurological manifestations without a clear-cut intestinal damage. The association between AGA and neurological manifestations has been referred to as gluten-sensitive ataxia / neuropathy (10, 15). AGA can cross react with epitopes expressed by Purkinje cells (16), thus expanding the spectrum of autoimmune related mechanism operating in neurological gluten-induced disorders.

Finally, neurological symptoms (i.e. headache, foggy mind, leg / arm numbness, depression) are also integral part of the clinical repertoire of non celiac gluten sensitivity (NCGS), a further manifestation of gluten-related disorders, and in this condition AGA have been identified in as many as 50% of patients (17,18). However, whether AGA contributes to the symptom generation in NCGS is still undeciphered and awaits further elucidation.

Patients with minor intestinal lesions (i. e. Marsh I and II) were not included in the present study. In fact, minor intestinal lesions can be expression of several pathological conditions including potential CD, NCGS, autoimmune disorders, immune-deficiency diseases, parasitic infections and food intolerance. Independently from diagnosis, a significant proportion of patients with normal villous architecture, but with mild duodenal inflammation, complain of bloating and alternating bowel habit ranging from diarrhea to constipation. Therefore, it would be interesting to evaluate the presence of NA, in particular to ENS, in patients with minimal lesions.

In conclusion, our study showed a high prevalence of NA to CNS in CD-related neurological disorders suggesting that these antibodies could be a marker of neurological involvement in CD. NA to ENS were associated with severe chronic constipation in neurological and non-neurological CD, thus leading to the concept of an underlying autoimmune mediated impairment of enteric neuron function. Taken together our data contribute to define the clinical and immunological features of NA to CNS and ENS in neurological as well as non-neurological CD and therefore pave the way for a better management of these patients.
